# White matter alterations and tract lateralization in children with dyslexia and isolated spelling deficits

**DOI:** 10.1002/hbm.24410

**Published:** 2018-09-29

**Authors:** Chiara Banfi, Karl Koschutnig, Kristina Moll, Gerd Schulte‐Körne, Andreas Fink, Karin Landerl

**Affiliations:** ^1^ University of Graz, Institute of Psychology Graz Austria; ^2^ Department of Child and Adolescent Psychiatry Ludwig‐Maximilians‐University Munich Germany; ^3^ BioTechMed‐Graz Graz Austria

**Keywords:** AFQ, dyslexia, isolated spelling deficit, structural asymmetry, tractography

## Abstract

The present study investigated whether children with a typical dyslexia profile and children with isolated spelling deficits show a distinct pattern of white matter alteration compared with typically developing peers. Relevant studies on the topic are scarce, rely on small samples, and often suffer from the limitations of conventional tensor‐based methods. The present Constrained Spherical Deconvolution study includes 27 children with typical reading and spelling skills, 21 children with dyslexia and 21 children with isolated spelling deficits. Group differences along major white matter tracts were quantified utilizing the Automated Fiber Quantification software and a lateralization index was calculated in order to investigate the structural asymmetry of the tracts. The two deficit groups mostly displayed different patterns of white matter alterations, located in the bilateral inferior longitudinal fasciculi, right superior longitudinal fasciculus, and cingulum for the group with dyslexia and in the left arcuate fasciculus for the group with isolated spelling deficits. The two deficit groups differed also with respect to structural asymmetry. Children with dyslexia did not show the typical leftward asymmetry of the arcuate fasciculus, whereas the group with isolated spelling deficits showed absent rightward asymmetry of the inferior fronto‐occipital fasciculus. This study adds evidence to the notion that different profiles of combined or isolated reading and spelling deficits are associated with different neural signatures.

## 1 INTRODUCTION

Cognitive models of reading (Coltheart, Rastle, Perry, Langdon, & Ziegler, 2001; Perry, Ziegler, & Zorzi, 2007) describe two main reading routes: The sublexical route, based on grapheme‐phoneme conversion rules for decoding unknown words and pseudowords and the lexical route, accessing the orthographic long‐term memory to read known regular or irregular words. Against this theoretical background, a dual‐route model for structural white‐matter correlates of reading was suggested by Vandermosten, Boets, Wouters, and Ghesquière (2012), incorporating a dorsal phonological and a ventral orthographic pathway. Based on the anatomical location of the two routes, Vandermosten, Boets, Wouters, and Ghesquière (2012) hypothesized that the dorsal route might involve the arcuate fasciculus (AF) and the superior longitudinal fasciculus (SLF), while the ventral route might overlap with the inferior fronto‐occipital fasciculus (IFOF) and the inferior longitudinal fasciculus (ILF). Correlational findings support this putative anatomical distinction. The rate of fractional anisotropy (FA) development over time in the left AF and left ILF was shown to be related to reading measures in English‐speaking children (Wang et al., 2017; Yeatman, Dougherty, Ben‐shachar, & Wandell, 2012). Similar correlational evidence was provided, for example, by Thiebaut de Schotten et al. (2014), who reported a positive association between FA in the posterior part of the left AF and reading performance in a group of Brazilian literate and illiterate adults. A recent longitudinal study (Myers et al., 2014) showed that FA in two left temporo‐parietal regions measured in kindergarten predicted reading performance in Grade 3. The observed clusters contained white matter of the AF, thus confirming its involvement in the reading network. Further evidence supports the dual‐route hypothesis that dorsal and ventral reading‐related tracts are functionally related to different reading strategies (e.g., sublexical and lexical). As part of the dorsal route for phonology‐based reading, the left AF and SLF were associated with performance on phonological awareness (PA) tasks (Saygin et al., 2013; Travis, Adams, Kovachy, Ben‐Shachar, & Feldman, 2017; Vandermosten et al., 2012; Yeatman et al., 2011). As part of the ventral route for lexical reading strategies, the left IFOF was related to orthographic processing (Gebauer, Fink, Filippini, et al., 2012; Vandermosten, Boets, Poelmans, et al., 2012). The corpus callosum (CC) is thought to play an important role in reading as well, since it supplies the interhemispheric connection of language‐specialized centers in the right and left hemispheres (Wandell & Yeatman, 2013). Indeed, associations between reading measures and white matter in the corpus callosum were reported (Dougherty et al., 2007; Lebel et al., 2013).

Structural white matter findings in dyslexia are rather heterogeneous. Meta‐analytic findings by Vandermosten, Boets, Wouters, and Ghesquière (2012) reported two clusters of reduced FA in poor compared with good readers: The bigger one was located in the left temporo‐parietal cortex and the smaller one in the proximity of the left inferior frontal gyrus. Fiber tracking revealed that some of the fibers in the bigger cluster belonged to the AF. Consistently, tractography studies confirmed such evidence showing reduced FA in the left AF in children with dyslexia (Christodoulou et al., 2017; Zhao, Thiebaut de Schotten, Altarelli, Dubois, & Ramus, 2016). Other studies, however, report lower FA and axial diffusivity (AD) in individuals with dyslexia compared with typical readers in widespread ventral and dorsal clusters, mostly in the left but also in the right hemisphere (Moura et al., 2016; Richards et al., 2008, 2015).

The heterogeneity of findings probably reflects differences in the analysis approach of diffusion weighted data and in selection criteria for dyslexia between studies. An important issue may be, whether or not participants experienced problems in spelling as well. Indeed, reading performance is usually taken as central criterion for a dyslexia diagnosis. Spelling performance is often only marginally considered, or it is merged with reading into a general composite score (Vanderauwera, Wouters, Vandermosten, & Ghesquière, 2017; Vandermosten, Boets, Poelmans, et al., 2012). It is at least surprising that spelling is sometimes not even reported (Christodoulou et al., 2017)

Our current knowledge of spelling‐related white matter networks is very limited and specific hypotheses are lacking. Functional MRI studies point toward a partial overlap between reading and spelling processes on the neuro‐functional level (Purcell, Jiang, & Eden, 2017; Rapp & Dufor, 2011; Rapp & Lipka, 2011). Therefore, it seems reasonable to assume that the structural brain substrates may be shared between the two literacy processes. One important way to identify the white matter tracts related to spelling is to investigate individuals with isolated spelling disorder (SD) in the context of adequate reading skills. Up to date, only two earlier studies with 11 and 19 German‐speaking children with SD are available. Gebauer, Enzinger, et al. (2012) found no structural white matter differences compared with a typically developing control group, while Gebauer, Fink, Filippini, et al. (2012) identified several clusters of reduced FA in the right hemisphere. They were interpreted as less efficient connectivity in right white matter pathways, most likely related to functional over‐activity in right‐hemisphere regions, mirroring inefficient cognitive compensatory strategies (Gebauer et al., 2012). This preliminary evidence is interesting, and it will be important to replicate the findings with a larger sample.

The present study investigated alterations in reading‐related white matter tracts in reasonably large and carefully screened children with dyslexia and isolated spelling deficits. Participants were recruited at the end of Grade 3. After 3 years of formal instruction, children are expected to read fluently and be familiar with the central aspects of orthographic spelling in German. In our study, we specifically addressed the question, whether children with dyslexia and children with SD would show different patterns of white matter alteration compared with a group of children with typical development. Eye‐tracking studies in German‐speaking children (Gangl, Moll, Banfi, et al., 2018; Gangl, Moll, Jones, et al., 2018) showed that dysfluent readers experience weaknesses in lexical as well as sublexical processes. In the present study, we thus expect to observe white matter alterations in both dorsal and ventral routes in the dyslexia group.

The mechanisms underlying written language processing in children with SD are less clear. Frith (1980) argued that children with SD apply partial cue reading strategies, without paying attention to the letter‐to‐letter structure of words. As a consequence of their poor sublexical skills, they do not develop well‐specified orthographic representations, which explains their poor spelling skills. Accordingly, Frith (1980) reported poor nonsense word reading in SD children, confirming her hypothesis of weak sublexical skills in this group. This finding, although interesting, was not replicated in more recent studies with German‐speaking children (Gangl, Moll, Banfi, et al., 2018; Moll & Landerl, 2009). Most likely due to the high grapheme–phoneme consistency in German, SD children's performance in word and nonword reading tasks was similar to typically developing readers, indicating unaffected accuracy and fluency. Thus, the question arises, whether or not children with SD show specific alterations in their white matter tracts compared with children with typical development as well as dyslexia. The present structural imaging study aims to provide further insights on processing strategies in SD children. If children with SD rely on degraded orthographic representations, which suffice for reading but not for spelling as suggested by Frith (1980), they are expected to show structural alterations in the ventral route for orthographic processing.

Phonological awareness is a well‐established predictor of literacy attainment across orthographies, with a stronger association with reading accuracy and spelling than with reading fluency (Moll et al., 2014). Accordingly, PA deficits have been reported in dyslexia as well as SD samples (Wimmer & Mayringer, 2002). In this study, we thus expect PA deficits to associate with spelling in both deficit groups. Lower FA should be evident in both deficit groups in phonology‐related white matter tracts, like the left AF and SLF. Finally, we expect to find structural alterations in the right hemisphere as an indicator of reduced connectivity of right white matter tracts in the SD group, as previously shown by Gebauer, Fink, Filippini, et al. (2012).

White matter characteristics were assessed by means of “Automated Fiber Quantification” (AFQ; Yeatman, Dougherty, Myall, Wandell, & Feldman, 2012), which represents a new tractography‐based approach for the analysis of diffusion‐weighted imaging data. It overcomes some of the limitations of previous methods. For example, classical tensor‐based methods yielded spurious results of reduced FA in regions of crossing fibers, such as the temporo‐parietal region where individuals with dyslexia usually display lower FA than typical readers and spellers. The anatomical localization of the clusters is also quite imprecise with such methods, because they usually rely on the coordinate match from probabilistic white matter atlases and thus cannot accurately differentiate tracts that run close to each other as the superior longitudinal fasciculus (SLF) and the AF or the ILF and the IFOF. Tractography is considered the most precise method to identify white matter tracts in vivo, but it is usually time‐consuming, requiring the manual delineation of regions of interest by expert anatomists. Diffusion properties are averaged along the entire tract, thus loosing levels of complexity as well as information that might be visible only from a more fine‐grained perspective. AFQ provides tract profiles that contain more information than standard diffusion measures. It returns tensor‐based parameters for 100 equidistant segments along 20 right and left white matter tracts with an automatic algorithm, thus improving the level of detail of the analysis.

Note that in the present study, the AF and SLF were treated as separate tracts and group comparisons were computed for each of them, separately. As pointed out by Zhao et al. (2016), most studies on reading and dyslexia did not consider the AF and SLF concurrently. This is a remarkable limitation for comparing findings. Although the anatomical description and differentiation of the two tracts is still under debate (Dick & Tremblay, 2012), they connect different brain regions and are thus likely to play different roles in reading and dyslexia.

We were also interested in potential alterations in the lateralization of reading‐related white matter tracts. Structural asymmetries in the brain are known to characterize the normal population, as for example the leftward asymmetry of the planum temporale, which was observed in 65% of the sample in the pioneer work by Geschwind and Levitsky (1968). Notably, subjects with dyslexia were reported to have a rather symmetric planum temporale in the post‐mortem investigation by Galaburda, Sherman, Rosen, Aboitiz, and Geschwind (1985). They suggested that this high prevalence of gray matter symmetry might result from deficient pruning mechanisms in subjects with dyslexia, most likely deriving from prenatal testosterone influences. This and further studies in the field were criticized due to the presence of confounding variables such as gender, IQ and handedness (Eckert & Leonard, 2000). However, recent studies controlling for such confounding factors replicated the evidence of reduced or absent leftward symmetry of the planum temporale in dyslexia (Altarelli et al., 2014) and in children at family risk for dyslexia (Vanderauwera et al., 2018). Structural asymmetries in the white matter were reported as well. The AF was shown to be left‐lateralized in adults and children (Catani et al., 2007; Lebel & Beaulieu, 2009; Qiu, Tan, Siok, Zhou, & Khong, 2011; Vandermosten, Poelmans, Sunaert, Ghesquière, & Wouters, 2013; Yeatman et al., 2011; Zhao et al., 2016). Its left‐lateralization was positively correlated with language‐related skills such as word learning (Catani et al., 2007), receptive vocabulary, phonological processing (Lebel & Beaulieu, 2009), and was associated with better reading scores (Qiu et al., 2011; but see Yeatman et al., 2011 for a negative correlation with reading). As Zhao et al. (2016) pointed out, dyslexia‐related deviant lateralization of gray matter structures should be associated with deviant white matter lateralization. Indeed, decreased left‐lateralization of a temporo‐parietal region corresponding to the centrum semiovale and superior corona radiate (Niogi & McCandliss, 2006) and the arcuate fasciculus (Vandermosten et al., 2013; Zhao et al., 2016) was reported in dyslexia. Zhao et al. (2016) found decreased leftward asymmetry of the IFOF and increased rightward asymmetry of the SLF in children with dyslexia. Vandermosten et al. (2013) reasoned that the leftward lateralization of the AF could be crucial for processing phonological information and the decreased or absent lateralization of this tract in dyslexia might be an indicator for a less developed neural network for reading. Accordingly, recent models suggest that, during development, the functional language network evolves from the reliance on bottom‐up processes, which are related to bilateral hemispheric activity, to top‐down processes that are functionally and structurally associated to the left hemisphere (Perani et al., 2011; Skeide & Friederici, 2016). In the present study, the degree of structural white matter asymmetry was analyzed in order to investigate whether altered tract lateralization is a specific feature related to dyslexia or whether it characterizes white matter organization of children with isolated spelling disorder as well.

## 2 METHODS

### 2.1 Compliance with ethical standards

The study was performed in accordance with the latest version of the Declaration of Helsinki and in compliance with national legislation. It was approved by the ethics committee of the University of Graz (Austria). Written informed consent was obtained on behalf of the children from their parents.

### 2.2 Participants and psychometric assessment

As the Austrian school system does not recognize any formal dyslexia diagnosis, the participants of the present study were recruited based on an extensive classroom screening with 2,562 children at the end of third or beginning of fourth Grade. Standardized classroom tests of sentence reading fluency (Wimmer & Mayringer, 2014) and spelling (Müller, 2004) as well as an individually administered standardized 1‐min word and pseudoword reading speed test (Moll & Landerl, 2010) were carried out in school. From this large sample, we recruited three groups:

1. Children with typical reading and spelling skills (*N* = 27) had percentiles between 25 and 85 on the mean of the three reading measures and on spelling.
2. For the dyslexia group we selected 20 children with serious problems (percentile ≤16) on two reading measures (and below average performance with a percentile not higher than 43 on the third reading measure). Three further children were admitted to this group. Two of them had a percentile of only 11 on one reading subtest and ≤20 on the two others, one child had percentile 16 in one reading subtest and ≤18 on the two others, also indicating serious problems with reading.
3. For the group with isolated SD, we selected 14 children with poor spelling performance (percentile ≤16) and age‐adequate reading (percentile ≥25 on the mean of the three reading measures). We admitted additional 7 children to this group who just about missed the spelling criterion by committing only one or two spelling errors less than the others (5 children had a spelling percentile of 17 and 2 had a percentile of 20). Importantly, these children also showed a very clear discrepancy between reading and spelling, with a mean reading percentile ≥30.

All children had German as their first language, a non‐verbal IQ ≥ 85 (Weiß, 2006), normal or corrected‐to‐normal vision, no identified sensory or neurological deficits, no clinical ADHD diagnosis as well as an above‐threshold score on a standardized parental questionnaire for attention deficits (Döpfner, Görtz‐Dorten, Lehmkuhl, Breuer, & Goletz, 2008). Children were also given the Vocabulary, Digit Span, and Symbol Search subtests of the Wechsler Intelligence Scale for Children (Petermann & Petermann, 2011). They performed a phoneme deletion task developed in our laboratory and standard paradigms of RAN‐objects and RAN‐digits (Denckla & Rudel, 1976). A full description of the literacy and cognitive measures is provided in the supplementary materials (Supporting Information Appendix S1).

Altogether 71 children were assessed. Two participants from the dyslexia group were excluded because more than five tracts could not be identified by the automatic algorithm, due to excessive movement artifacts in the anatomical T1 images. Because of drop out during the last behavioral assessment, data on RAN were not available for two children with dyslexia.

Table 1 reports descriptive statistics for each literacy and cognitive measure. The three groups did not differ with respect to gender χ(2) = 1.43, *p* = .490 and handedness χ(2) = .24, *p* = .886. The significant age difference reported in Table 1 is due to the fact that the dyslexia group was about 4 months younger than the SD group, *p* = .051, with no significant differences of the dyslexia and SD groups relative to typical readers and spellers (*p*s > .250). Table 1 shows seriously impaired performance with mean percentiles around 10 for reading in the dyslexia group and just above percentile 10 for spelling in the dyslexia and SD groups. Children of the dyslexia group showed lower performance than the typical and SD groups in sentence, word and pseudoword reading, whereas the SD group had age‐adequate reading skills, which did not differ from the typical group. Both deficit groups showed clearly lower spelling percentiles than typical readers and spellers, they did not differ in the severity of their spelling impairment.

**Table 1:** Mean scores (*M*) and standard deviations (*SD*) for age, literacy, and cognitive measures in the three groups

	Typical			Dyslexia			SD					
	*n* = 27			*n* = 21			*n* = 21					
	*M*		SD	*M*		SD	*M*		SD	F	p	ES
*N* (males)	27 (15)			21 (12)			21 (15)					
Right handed	24			18			19					
Age in months	113.00		3.61	111.67	^3^	4.53	115.71	^2^	7.58	3.13	.050	.087
Sentence reading percentile (SLS)	55.48	^2^	17.51	10.76	^1^^,^^3^	8.71	54.62	^2^	15.56	66.17	<.001	.667
Word reading percentile (SLRT‐II)	49.33	^2^	13.52	9.43	^1^^,^^3^	6.57	48.48	^2^	19.72	56.25	<.001	.630
Pseudoword reading percentile (SLRT‐II)	54.63	^2^	14.76	10.81	^1^^,^^3^	7.32	50.05	^2^	23.09	48.90	<.001	.597
Spelling percentile (DRT‐3)	50.63	^2^^,^^3^	12.70	16.67	^1^	10.99	12.24	^1^	5.04	100.69	<.001	.753
Nonverbal IQ	103.93		9.93	98.90		9.91	100.19		9.37	1.75	.183	.050
Vocabulary standard score (WISC‐IV)	11.67		3.15	10.76		2.43	10.33		3.07	1.32	.275	.038
Digit span standard score (WISC‐IV)	10.59		2.86	11.43		2.60	9.81		2.20	2.04	.138	.058
Symbol search standard score (WISC‐IV)	11.44		1.80	11.38		2.09	11.76		2.28	.21	.808	.006
Phonological awareness (% correct)	.88	^2,3^	.09	.75	^1^	.13	.77	^1^	.13	9.63	<.001	.226
RAN digits/s	1.99	^2^	.31	1.60	^1^^,^^3^	.29	1.92	^2^	.49	6.56	.003	.170
RAN objects/s	1.08	^2^	.19	.93	^1^	.13	1.06		.17	4.48	.015	.123
ADHD questionnaire	.36	^3^	.29	.61		.44	.74	^1^	.41	6.08	.004	.155

*Note*. Subscripts indicate significant differences on post‐hoc analyses with Bonferroni correction for multiple comparisons, to: 1: Typical group, 2: Dyslexia group, 3: SD group. Effect sizes (*ES*) are calculated as partial eta‐squared.

There were no significant group differences on nonverbal IQ and all WISC subtests (Vocabulary, Digit Span and Processing Speed). Children with dyslexia showed the typical profile of impairment in both PA and RAN (Moll & Landerl, 2009). The SD group had lower scores than the typical group on the PA task, which is consistent with an earlier study by Wimmer and Mayringer (2002). Although children with high ADHD‐scores were not admitted to the study, we observed a significantly higher ADHD score (indicating more ADHD‐symptoms reported by parents) for the SD group compared with typical readers and spellers, whereas the dyslexia group did not differ from typical and SD groups.

### 2.3 Imaging acquisition

Imaging was performed on a 3.0 T Skyra scanner (Siemens Healthineers, Erlangen, Germany) using a 20‐channel head coil. High‐resolution 3D‐T1 MPRAGE (1 mm isotropic) structural scans (TR = 1,600 ms, TE = 1.79 ms) and multiband EPI DTI data (2.5 × 2.5 × 2.5 mm^3^ isotropic voxels, TR = 3,400 ms, TE = 105 ms, matrix 96 mm × 96 mm; FOV = 240 mm, flip angle: 86°; *b* value = 2,000 s/mm^2^, 1 × *B* = 0 images, 64 directions, 48 slices) were acquired. The overall scan time took about 12 min. To correct for susceptibility‐induced distortions, the same multiband sequence was collected with forward and reversed phase encoding blips.

### 2.4 Data preprocessing

As first step, diffusion‐weighted (DW) images were denoised with the “dwidenoise” command in the MRtrix package (J‐D Tournier, Brain Research Institute, Melbourne, Australia, https://github.com/MRtrix3/mrtrix3) (Tournier, Calamante, & Connelly, 2012). Eddy current‐induced distortion, motion and susceptibility‐induced distortion corrections were performed with “dwipreproc” in MRtrix, which relies on FSL (Jenkinson, Beckmann, Behrens, Woolrich, & Smith, 2012). A brain mask was applied with bet (Jenkinson et al., 2012). The B1 field inhomogeneity correction was performed with the “dwibiascorrect” command (Tustison et al., 2010). Anatomical T1 images underwent Bias Field correction with N4 (Tustison et al., 2010). Images were then segmented into five tissues using the “5ttgen” algorithm (Smith, Tournier, Calamante, & Connelly, 2012). The response function (RF) was estimated with “dwi2fod.” Based on the RF, Constrained Spherical Deconvolution data were obtained with the command “dwi2fod,” which allowed the computation of the fiber orientation distribution. A whole‐brain probabilistic tractography algorithm with 5 million tracks was then applied, which were reduced to 1 million by means of the spherical‐deconvolution informed filtering of tractograms algorithm (SIFT; Smith, Tournier, Calamante, & Connelly, 2013).

### 2.5 Tract quantification

Whole‐brain tractography data were imported into the AFQ software package (https://github.jyeatman/AFQ; Yeatman, Dougherty, Myall, et al., 2012) running on MATLAB (2015b, The MathWorks, Natick, MA). Tract diffusion profiles were obtained with “AFQ_run,” which returned a structured array containing tensor‐based measures of the 20 tracts for each group. Among other tensor‐based parameters, we focused specifically on fractional anisotropy (FA), which was calculated on 100 nodes in each delineated tract: right and left thalamic radiations, forceps major and minor of corpus callosum, right and left inferior fronto‐occipital, inferior longitudinal, superior longitudinal, arcuate and uncinate fasciculi, corticospinal tract, and cingulum.

### 2.6 Lateralization index

Lateralization was investigated for specific tracts of interest, known to play a role in reading from the literature (Vandermosten, Boets, Wouters, & Ghesquière, 2012; Wandell & Yeatman, 2013), naming the superior longitudinal, arcuate, inferior longitudinal and the inferior fronto‐occipital fasciculi. The lateralization index (LI) was calculated on FA as (R – L)/(R + L), thus yielding positive values for right‐lateralized and negative values for left‐lateralized tracts.

## 3 RESULTS

### 3.1 Tract quantification

Tract profiles and lateralization indexes were computed for the remaining 69 participants. The tractography algorithm could identify 64 tract profiles for the forceps major of corpus callosum, 68 tract profiles for the left and right cingulum, 67 tract profiles for the left uncinate fasciculus, 55 for the left IFOF, and 50 for the right IFOF. The reduced number of children for whom the lateralization index could be calculated for the IFOF was thus reduced to 44. Some nodes of the left AF, left and right SLF, cingulum, and uncinate fasciculi could not be quantified in all participants, *n* thus varied between 66 and 69.

### 3.2 Group comparison on the tracts

Mean FA values of the WM fiber tracts were generally normally distributed, with the exception of the right corticospinal tract, forceps major of corpus callosum, left IFOF, right SLF, and left uncinate fasciculus (as assessed by the Kolmogorov–Smirnov test). For control purposes, non‐parametric tests were conducted for variables deviating from normality, and yielded a pattern of results similar to the parametric ANOVAs. Results on parametric tests are thus reported. Although groups did not differ significantly with respect to the motion parameter [Euclidian distance of *x*, *y*, and *z* translation; Tromp (2016)], *F*(2,68) = 0.61, *p* = .545, it was included as a covariate in the analysis to control for slight changes that might influence group differences. To avoid reporting false positives due to the high number of comparisons performed, significant group differences at *p* ≤ .05 were reported only if they encompassed more than three adjacent nodes. To further prevent the occurrence of type I errors, the significance level of *p* = .05 was divided by three, that is, the number of group comparisons performed on each node. Results are thus reported at the more stringent significance level of *p* ≤ .017, as well as at the more lenient one of *p* ≤ .05. A summary of all significant group differences for each node of the respective fiber tract is given in Supporting Information Table S1.

**Figure 1:**
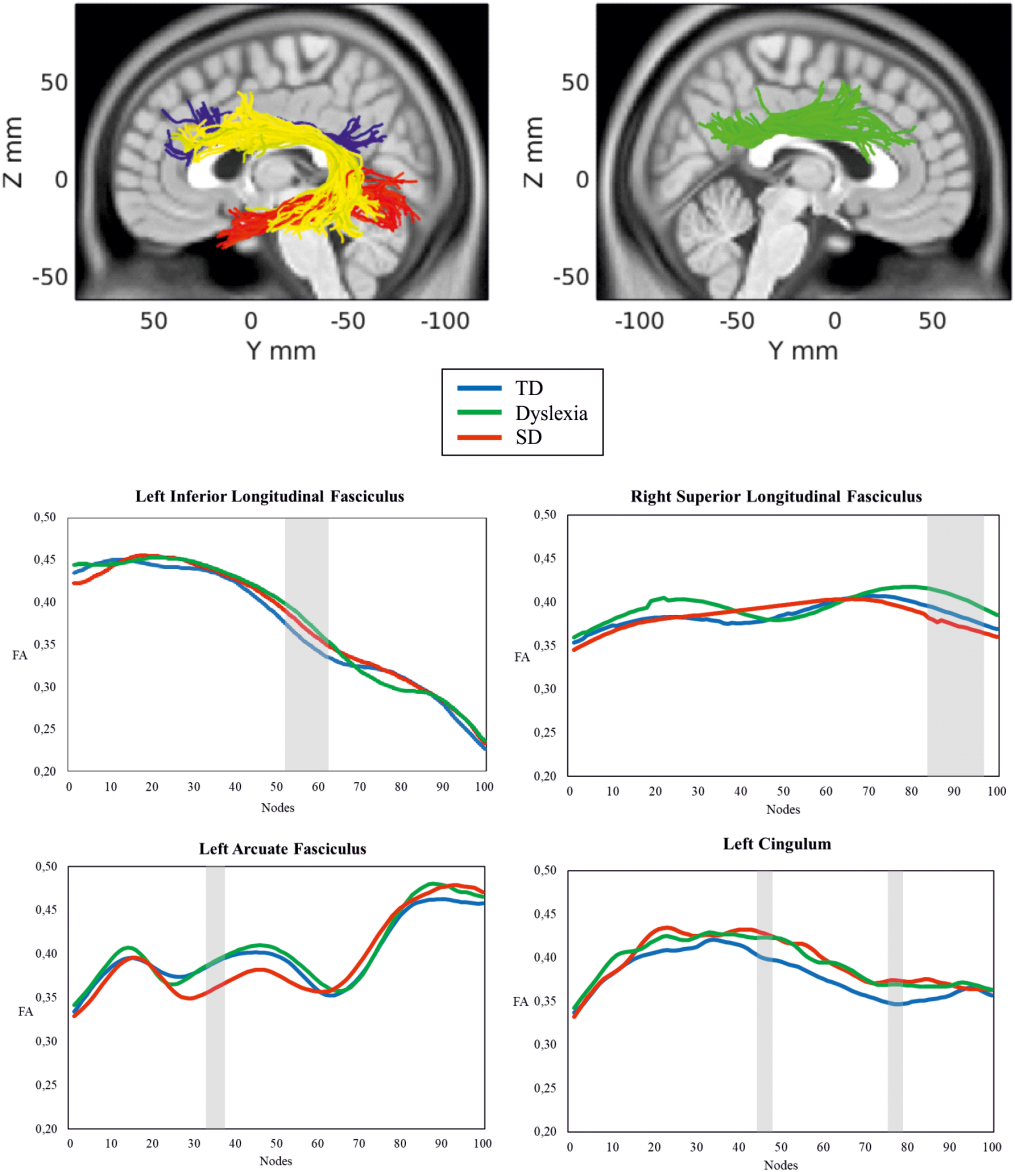
Panel above: Anatomical rendering of the left inferior longitudinal fasciculus (red), right superior longitudinal fasciculus (green), left arcuate fasciculus (yellow), and left cingulum (blue). Panels below: Tract profiles for the three groups (blue: Typical readers and spellers; green: Dyslexia group; red: SD group). The gray‐shadowed areas highlight regions on the tracts where groups differed. Nodes are ordered in the anterior–posterior direction for the left arcuate fasciculus, in the posterior–anterior direction for the inferior, superior longitudinal fasciculi and the cingulum [Color figure can be viewed at http://wileyonlinelibrary.com]

As shown in Figure 1 (see also Supporting Information Table S1 and Supporting Information Figure S1), the overall pattern of results suggests a quite different picture of increases and decreases in FA in the two deficit groups compared with typical readers and spellers. Unexpectedly, the dyslexia group showed no clusters of selective FA reductions in comparison to the other two groups. Instead, children with dyslexia displayed higher FA in the left ILF (nodes 52–61), right ILF (nodes 1–6), right SLF (nodes 83–97), and right cingulum (nodes 7–10). The SD group showed lower FA in the left arcuate fasciculus (nodes 32–37) compared with the TD group.

A similar pattern of white matter alterations in the two deficit groups was observed for the left cingulum (nodes 45–48, 76–79). However, only the SD group showed significantly higher FA than the TD group in both clusters (see Supporting Information Figure S1).

In order to rule out the possibility that structural differences between the dyslexia and SD groups could have been driven by their small but significant age difference, ANCOVAs were re‐run with age as a covariate. This had no significant impact on the results. On the basis of these findings we can conclude that the small age lag between the dyslexia and the SD groups had no substantial impact on the main pattern of our results.

### 3.3 Association of tract nodes with literacy variables

**Figure 2:**
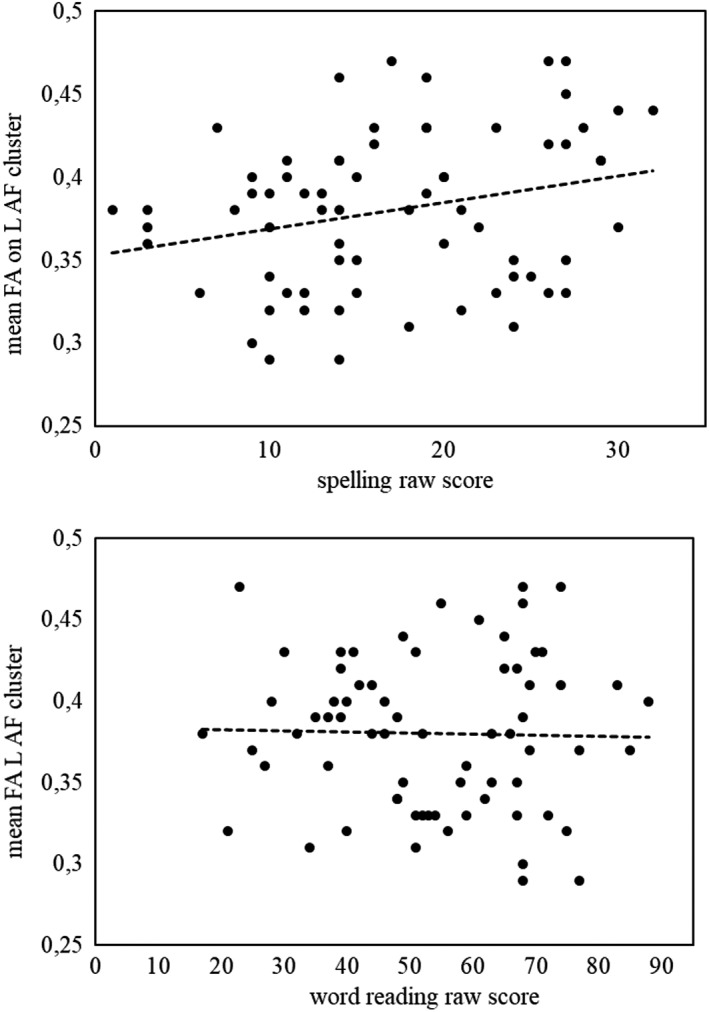
Panel above: Scatterplot presenting significant and positive correlation between FA on the left arcuate fasciculus and spelling. Panel below: Scatterplot presenting absent correlation between FA on the left arcuate fasciculus and word reading

To further examine whether the observed group differences were specific to reading or spelling, partial correlations were calculated between raw scores on literacy measures and the mean FA in each cluster where group differences emerged. To correct for multiple comparisons, the significance level of .05 was divided by 2, as two constructs were correlated to each FA cluster (reading and spelling). We thus consider a value of *p* = .025 as the threshold for significant correlations. The dyslexia group showed higher FA in the right ILF. Accordingly, significant negative correlations were observed between the right ILF and reading measures, controlling for spelling (*r* = −.30, *p* = .013 for word reading; *r* = −.31, *p* = .011 for pseudoword reading), indicating that better reading outcomes were associated to lower FA. Note that the association between FA in this cluster and spelling was not significant (*r* = −.08, *p =* .52). The SD group showed lower FA in the left AF compared with typical readers and spellers. Mean FA in this cluster was positively correlated with the spelling, controlling for reading measures (*r*s ≥ .28, *p* ≤ .019). Note that the association of this cluster with reading measures was not significant (*r*s between −.003 and −.19, *p* > .108). Figure 2 reports scatterplots showing the selective association of FA in the left AF cluster with spelling but not with reading. Finally, the SD (and dyslexia) groups displayed higher FA in two clusters on the left cingulum compared with typical readers and spellers. Mean FA in the first cluster (nodes 45–48) was significantly and inversely correlated with spelling, controlling for reading (*r* = −.29, *p* = .017). Again, correlations with reading were not significant (*r*s between −.11 and .02, *p* > .362).

### 3.4 Lateralization index (LI)

**Figure 3:**
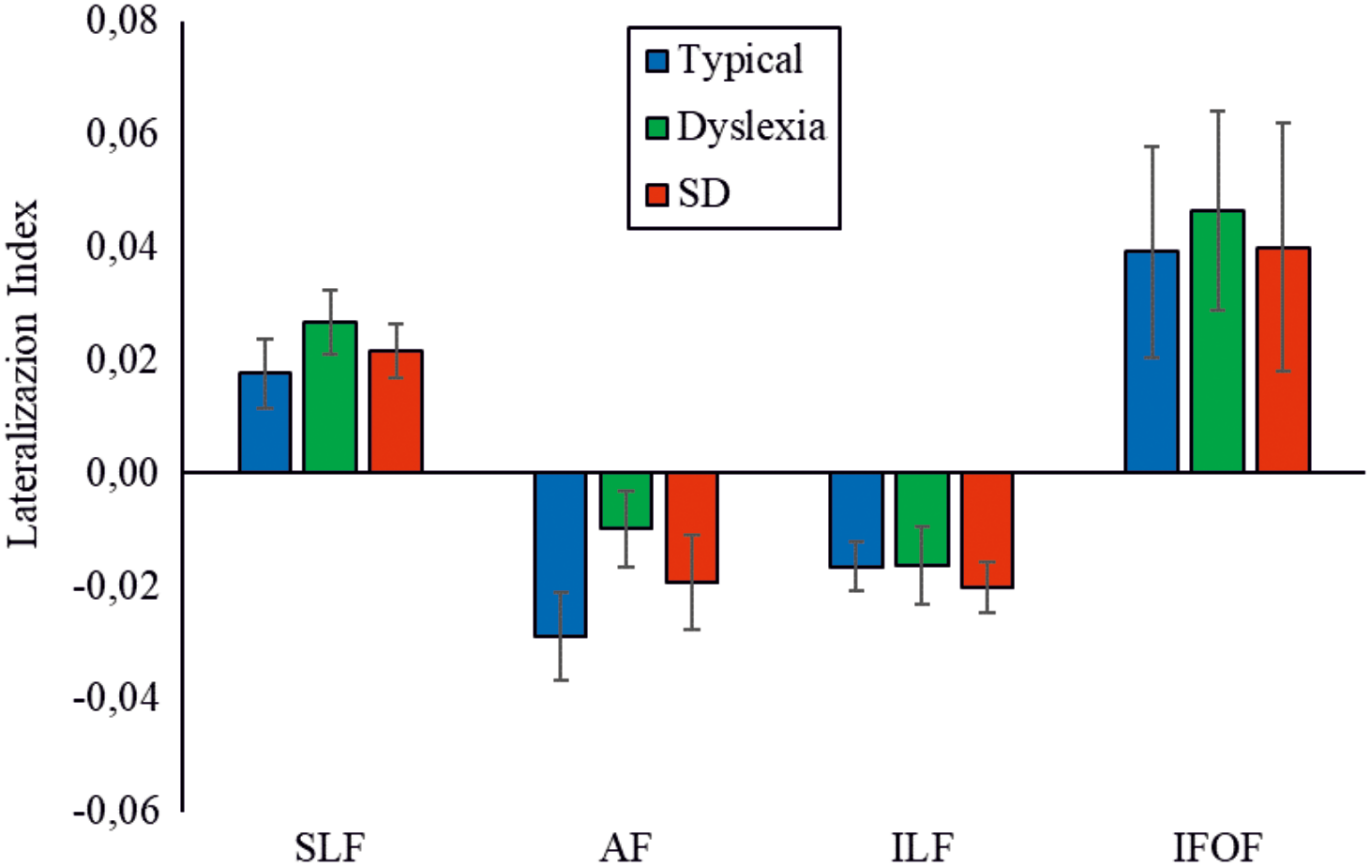
Laterality index in the three groups. Bars represent standard errors. SLF, Superior longitudinal fasciculus; AF, Arcuate fasciculus; ILF, Inferior longitudinal fasciculus; IFOF, Inferior fronto‐occipital fasciculus [Color figure can be viewed at http://wileyonlinelibrary.com]

The lateralization indexes of the four tracts were normally distributed (as assessed by means of the Kolmogorov–Smirnov test). As shown in Table 2 and Figure 3, the SLF and IFOF were lateralized to the right in all three groups, whereas the AF and ILF were lateralized to the left. One‐sample *t*‐tests against zero confirmed the significance of the corresponding pattern of lateralization. The LI of the AF was not significantly different from zero in the dyslexia group, whereas the LI of the IFOF was not significantly different from zero in the SD group, showing no clear lateralization of these tracts in the deficit groups.

**Table 2:** Means (*M*) and standard deviations (*SD*) for the lateralization index in the four tracts for each group, with *t* and *p* values of the one‐sample *t*‐test against zero

	TD					Dyslexia				SD					
	*M*	*SD*	*t*	*p*	*ES*	*M*	*SD*	*t*	*p*	*ES*	*M*	*SD*	*t*	*p*	*ES*
LI SLF	.02	.03	2.92	.007	.667	.03	.03	4.76	<.001	1.00	.02	.02	4.50	<.001	1.00
LI AF	−.03	.04	−3.72	<.001	.750	−.01	.03	−1.48	.155	.333	−.02	.04	−2.33	.030	.500
LI ILF	−.02	.02	−3.83	<.001	1.00	−.02	.03	−2.37	.028	.667	−.02	.02	−4.47	<.001	1.00
LI IFOF	.04	.08	2.08	.054	.500	.05	.06	2.63	.022	.833	.04	.08	1.82	.093	.500

*Note*. Effect sizes (*ES*) are calculated as Cohen's *d*.

### 3.5 Association of Lateralization Index with literacy and cognitive variables

The association of structural lateralization of white matter tracts with literacy and cognitive variables was further investigated by means of Pearson correlations. To correct for multiple comparisons, the significance level of .05 was divided by 9, corresponding to the number of constructs that were correlated to each LI (reading, spelling, nonverbal IQ, Vocabulary, Digit Span, Symbol Search, PA, RAN digits and RAN objects, ADHD score). We thus consider a value of *p* = .006 as the threshold for significant correlations. The only significant association emerged between LI of the AF and word reading, which were negatively correlated, *r*(67) = −.36, *p* = .002, suggesting that a more left lateralized pattern was associated to better reading skills.

## 4 DISCUSSION

The present study investigated differences in white matter between children with dyslexia and isolated spelling deficits in comparison to typically developing peers. While children with dyslexia showed the typical profile of reading impairments associated with significant deficits in PA and RAN, the group with isolated spelling deficit showed age adequate reading and no RAN deficit. Children in the two impaired groups exhibited serious deficits in reading and/or spelling, with mean percentiles of 10 on all three reading as well as the spelling tasks. Children with SD had spelling percentile just above 10, while their reading performance was within the normal range.

As expected, our findings revealed distinct patterns of structural alterations for the two deficit groups. Consistent with our hypothesis, the dyslexia group showed structural alterations within the dorsal and ventral routes, thus sustaining behavioral evidence reporting weaknesses in lexical and sublexical processes (Gangl, Moll, Banfi, et al., 2018; Gangl, Moll, Jones, et al., 2018). Compared with children with typical development, the dyslexia group displayed higher FA in the left ILF and right cingulum. Increased FA in the dyslexia group was also observed in the right ILF and SLF (with significant differences compared with the SD group). Mean FA on the right ILF correlated negatively with reading measures, controlling for spelling variance. Thus, poorer literacy performance was accompanied by higher FA, suggesting that the increase is in some sense dysfunctional. This finding is unexpected, as previous studies reported reduced FA in dyslexia and positive correlations with reading measures (Beaulieu et al., 2005; Deutsch et al., 2005; Lebel et al., 2013; Steinbrink et al., 2008). Recent tractography studies also showed lower FA in pre‐reading children at family risk for dyslexia (Langer et al., 2017; Vanderauwera et al., 2017; Vandermosten et al., 2015; Wang et al., 2017), which is again not in line with our findings. These studies, however, relied on different age groups, tractography algorithms and tract quantification techniques, which makes a direct comparison with the current findings rather difficult. Furthermore, most group differences on FA between good and poor readers were not significant (Vanderauwera et al., 2017) or were not even reported (Wang et al., 2017) once children carrying the family risk for dyslexia learnt to read.

Note that findings from tractography studies using AFQ software are mostly consistent with our findings in reporting negative correlations between FA and reading (Huber, Donnelly, Rokem, & Yeatman, 2018; Travis, Ben‐shachar, Myall, & Feldman, 2016; Yeatman, Dougherty, Ben‐shachar, & Wandell, 2012). Particularly interesting are findings from a longitudinal study by Yeatman, Dougherty, Ben‐shachar, and Wandell (2012): At the onset of the study, when children were aged 7–12, above‐average readers had lower FA than below‐average readers in the left AF and ILF. The pattern, however, changed during development: Above‐average readers tended to have a positive linear increase of FA over time, while below‐average readers showed a negative linear trend indicating a decrease in FA with age. As a consequence, 3 years later above‐average readers had higher FA than below‐average readers. Yeatman et al. suggested a dual process account of white matter development to explain their findings. Myelination and pruning would take place synchronously in above‐average readers, with FA values increasing monotonically with age. In below‐average readers the two processes would instead be asynchronous, with myelination preceding pruning and thus causing a decrease in FA in the later developmental stages. Our cross‐sectional data based on children in a similar age range provides some support for this hypothesis as we could confirm a negative correlation between reading performance and FA. Contrary to Yeatman et al., however, who investigated left hemisphere tracts, we observed negative reading‐related correlations in the right ILF.

Other studies also reported increased FA in left as well as right hemispheres in adults (Richards et al., 2008) and children (Rimrodt, Peterson, Denckla, Kaufmann, & Cutting, 2011) with dyslexia. However, these findings were either not discussed or related to a technical bias due to normalization. Note that in the current study we used technically advanced methods of tractography, which can be assumed to produce more reliable findings. Still, further studies will be needed to understand whether such left and right hemisphere increases in poor readers reflect divergent trajectories of FA development or should rather be considered as technical problems. A third possibility to explain this inconsistency may result from the heterogeneous manifestation of dyslexia. Indeed, white matter disorganization was related to genetically driven alteration of neural migration in dyslexia (Marino et al., 2014; see Mascheretti et al., 2017 for a recent review). In this perspective, inconsistencies in the literature on lower as well as higher FA (like in the present study) might simply mirror the heterogeneity of the biological manifestation of the disorder.

Contrary to our expectations of white matter alterations within the ventral route for orthography‐based processing, children with isolated spelling deficit showed a selective alteration in the left AF, where they displayed lower FA than the TD group. White matter in this tract was positively correlated with spelling but not with reading measures. The left arcuate fasciculus is generally assumed to be part of the dorsal phonological pathway and has been associated to PA performance (Saygin et al., 2013; Vandermosten, Boets, Poelmans, et al., 2012; Yeatman et al., 2011). Although the SD group had lower PA scores compared with the TD group, it is rather unlikely that differences in the left AF reflect such PA deficit in the SD group. Indeed, children with dyslexia had a similar PA deficit as the SD group, but did not show any cluster of lower FA in the left AF. Earlier studies found alterations in dyslexia in the left AF (Christodoulou et al., 2017; Vandermosten, Boets, Wouters, & Ghesquière, 2012; Wang et al., 2017). However, note that in some cases spelling performance was not even reported. Furthermore, FA in a left temporo‐parietal region most likely corresponding to the arcuate fasciculus was previously shown to be associated with spelling (Deutsch et al., 2005). Thus, in addition to the well‐documented association between the left arcuate fasciculus and reading, our findings suggest that alterations in this tract might also come into play in children with isolated spelling problems.

Our results do not confirm the presence of reduced FA in right hemisphere tracts in children with spelling disorder, as previously reported by Gebauer, Fink, Filippini, et al. (2012). One plausible explanation for this lack of consistency might be the different approaches to the diffusion‐weighted data. Gebauer, Fink, Filippini, et al. (2012) used a simple tensor model with 12 encoding directions to fit their data and the TBSS approach for the DTI analysis. This methodological choice might have limited the accuracy of the anatomical localization of the findings for crossing or intermixing fibers.

The only structural alteration that appeared similarly in both deficit groups (dyslexia as well as SD) compared with typically developing children was higher FA in the left cingulum. Although it was only significant for the contrast SD versus TD group in two specific clusters, this difference was evident on more than two thirds of the tract. White matter in the left cingulum has already been shown to be abnormal in dyslexia (Moura et al., 2016; Richards et al., 2015) and was suggested to relate to impaired executive functions among children with dyslexia and dysgraphia (Richards et al., 2015). However, in the present study mean FA on the left cingulum was selectively associated with spelling, but not reading. Although our findings are rather preliminary and need further replication, they suggest that alterations in the left cingulum might be specifically associated with spelling problems.

Both deficit groups showed comparable PA deficits at the behavioral level, but they did not display similar structural alterations on left dorsal phonological tracts. This finding is interesting, because it shows that a similar behavioral pattern does not coincide with a corresponding structural alteration. We might suggest, though speculatively, that PA weaknesses in the two deficit groups might have a different origin, as the two groups display rather different variation in white matter substrates.

To sum up, the dyslexia and SD groups showed different substrates for white matter alterations in comparison to typical readers and spellers. They were located in the bilateral ILF, right SLF and cingulum in the dyslexia group and in the left AF in the SD group. Both deficit groups showed higher FA than typical readers and spellers in the left cingulum. Our findings show selective associations of white matter with literacy measures. Although our results were not significant at very stringent significance thresholds, it is important to underline that they would not have been observed if FA had been averaged over entire tracts instead of being quantified on 100 nodes. Thus, the tractography method used in the current analysis provides us with additional quantitative and qualitative information on the tracts in the three groups assessed. That said, this tract quantification approach has been developed in very recent years and thus our findings should be interpreted with caution until replicated. We were not able to differentiate the three segments of the AF and SLF as shown by Catani, Jones, and Ffytche (2005) and Thiebaut de Schotten et al. (Thiebaut de Schotten et al., Dell'Acqua, 2011; Thiebaut de Schotten, Ffytche, et al., 2011). We thus cannot reason on possible distinct roles of tracts’ subparts. Furthermore, an important limitation of the present study could be also seen in the use of a tensor‐based parameter, that is, fractional anisotropy, to quantify fiber information obtained with Constrained Spherical Deconvolution tractography. Other parameters (e.g., HMOA as used by Zhao et al., 2016) may provide more precise information. A major challenge will hence be to find an appropriate parameter in AFQ, which will adequately quantify the very precise information on white matter structure obtained with Constrained Spherical Deconvolution tractography.

A further limitation of our study is that we could not rely on clinical diagnoses of a reading or spelling disorder, as such diagnoses are neither undertaken nor recognized by the Austrian school system. Note, however, that 90% of the dyslexia sample and about 70% of the SD sample fulfilled the criteria for a clinical diagnosis according to the German diagnostic guidelines (Galuschka & Schulte‐Körne, 2016).

### 4.1 Lateralization index

The present study investigated the structural lateralization of four dorsal and ventral white matter tracts involved in phonological and lexical processes (Vandermosten, Boets, Wouters, & Ghesquière, 2012; Wandell & Yeatman, 2013), namely, the SLF, AF, ILF, and IFOF. Two tracts were found to be consistently lateralized to the right in the three groups, that is, the SLF and the IFOF, whereas the AF and ILF showed leftward asymmetry. Our results are partially in line with previous tractography evidence, reporting rightward asymmetry for the second and third segments of the SLF (Thiebaut de Schotten, Dell'Acqua, et al., 2011; Zhao et al., 2016) and a leftward asymmetry for the long segment of the AF (Catani et al., 2007; Thiebaut de Schotten, Ffytche, et al., 2011; Zhao et al., 2016). As previously mentioned, the current study did not distinguish the sub‐components of AF and SLF. Our findings on laterality are thus not fully comparable to tractography studies which differentiated tracts' sub‐components. Note, however, that a leftward lateralization of the AF is in line with previous studies that did not differentiate the AF into the three sub‐parts (Lebel & Beaulieu, 2009; Qiu et al., 2011; Vandermosten et al., 2013; Yeatman et al., 2011). Furthermore, our study confirmed the association between the leftward asymmetry of the AF and better reading achievement in children (Qiu et al., 2011). Consistent with our findings, a leftward asymmetry of the ILF has already been described in adults (Thiebaut de Schotten, Ffytche, et al., 2011). This evidence, however, was not confirmed in a more recent tractography study on children (Zhao et al., 2016), where the ILF was not clearly lateralized toward one hemisphere. Finally, the finding of a rightward asymmetry of the IFOF is not in line with previous evidence in adults (Thiebaut de Schotten, Ffytche, et al., 2011) and children (Zhao et al., 2016). However, results are not fully comparable between studies, as different tractography methods and alternative approaches for the delineation of the tracts were applied. It is also important to note that the LI on the IFOF could be calculated for only 64% of the children in our sample, due to missing values in the tractography outcome for this tract.

We also observed interesting group differences: No clear lateralization was evident in the AF in the dyslexia group and in the IFOF in the SD group.

The present study replicates previous results of reduced lateralization of the AF in adults with dyslexia (Vandermosten et al., 2013). Our findings are also in line with Zhao et al. (2016), who reported a left lateralization of the posterior part of the AF in typical readers and no clear lateralization of the same portion of the tract in children with dyslexia. Interestingly, recent evidence suggests that altered structural lateralization might precede reading instruction and—perhaps—play a causal role in the development of reading impairments. Indeed, Vanderauwera et al. (2018) showed atypical asymmetry of the Planum Temporale in young pre‐readers with family history for dyslexia, thus observing structural alterations before literacy instruction. Similarly, Wang et al. (2017) reported altered lateralization of the AF in children with family history of dyslexia in pre‐ and beginning reading stages. It would be of great interest for further longitudinal studies to consider whether good and poor readers with a family history of dyslexia (and thus carrying the biological risk) still differ in the lateralization of white matter tracts once they enter the fluent reading stage.

The SD group did not show the expected rightward lateralization of the IFOF, which was present in typical and the dyslexia groups. This tract was suggested to constitute the ventral stream for orthographic processing of written material (Vandermosten, Boets, Wouters, & Ghesquière, 2012), which would explain why we found this alteration in asymmetry among children with selective orthographic difficulties as reflected in their poor spelling. As pointed out above, however, the bilateral IFOF was available for only 64% of the sample, due to reconstruction problems in AFQ. We would thus interpret this finding carefully, due to the restricted sample size.

## 5 CONCLUSION

The present findings underline the importance of considering spelling as well as reading measures in structural (and functional) imaging investigations of individuals with dyslexia and, more generally, literacy disorders. In our sample, children with dyslexia showed different structural alterations compared with their peers with isolated spelling deficits. Our results add thus further evidence that different cognitive and neural impairments may underlie selective profiles of combined or isolated reading and spelling deficits.

## Supporting information


**Appendix S1:** Supporting Information

**Supplementary Figure S1:** Tract profiles for the right inferior longitudinal fasciculus and cingulum in the three groups (blue: Typical readers and spellers; Green: Dyslexia group; Red: SD group). The gray‐shadowed areas highlight regions on the tracts where groups differed. Nodes are ordered in the posterior–anterior direction

**Supplementary Table S1:** Nodes on white matter tracts were groups differed on FA. Results at the more stringent significance level of *p* ≤ .017 are reported in the upper part of the table. Results at *p* ≤ .05 are reported in the bottom part of the table. Means (*M*) and standard deviations (*SD*) on FA are reported, with the statistical significance of relevant Bonferroni‐corrected post‐hoc comparisons.
