# Supplementary material for: White matter alterations and tract lateralization in children with dyslexia and isolated spelling deficits
Source: Hum Brain Mapp. 2018 Sep 29;40(3):765–76. doi: 10.1002/hbm.24410 (PMC6492145; doi:10.1002/hbm.24410)
Supplement: Supplementary file 1 — Appendix S1: Supporting Information [file HBM-40-765-s001.docx]

**Supplementary Appendix S1**

**Tasks**

The tasks described in this paper were part of a larger task battery comprising an initial screening in school including the classroom measures of sentence reading and spelling and the nonverbal IQ test. The individually administered word and pseudoword reading test was given in school as well. All other tasks were carried out as part of three to four individual assessments in our lab, each of which lasted between 90 and 120 minutes. The tasks described here were usually carried out during the first and second assessments, which took place between 2 to 12 weeks after the classroom screening.

**Reading**. In the classroom-administered standardized reading speed test (Wimmer & Mayringer, 2014, parallel test reliability is .95 for Grade 2 and .87 for Grade 8), children were asked to silently read single-line-long sentences with simple semantic and syntactic structure (e.g., “Trees can speak”); They had to mark each sentence as right or wrong by circling a check mark or a cross at the end of the line. The task was terminated after three minutes. The raw score was the number of correctly marked sentences.

In the individually administered one-minute reading speed test (Moll & Landerl, 2010, parallel test reliability is .94 for words and .90 for pseudowords), children were instructed to read aloud a word and a pseudoword list as fast as possible without making errors. The number of correctly read items within one minute was taken as raw score.

**Spelling**. The standardized classroom spelling task (Müller, 2004; split-half reliability is .95) comprised 44 words that had to be written into sentence frames. The experimenter dictated each word, then read out the full sentence and then repeated the word again. The number of correct word spellings was scored.

**Nonverbal IQ.** The first part of the German version of the Culture Fair Intelligence Test (Weiß, 2006; test reliability = .92 according to manual) was given as an estimate of nonverbal IQ. Its four subtests comprised Series, Classification, Matrices and Topology.

**Vocabulary** was assessed by the vocabulary subtest of the German version of the Wechsler Intelligence Scale for Children (Petermann & Petermann, 2011).

**Verbal short-term and working memory** were investigated by the Digit Span subtest of the German version of the Wechsler Intelligence Scale for Children (Petermann & Petermann, 2011).

**Speed of processing** was investigated by the Symbol Search subtest of the German version of the Wechsler Intelligence Scale for Children (Petermann & Petermann, 2011).

**Phonological awareness (PA)** was assessed by means of a computerized phoneme deletion task running on Presentation 16.3 (Neurobehavioral Systems, Inc., Berkeley, CA, USA). The task consisted of four practice trials and 25 test trials (20 mono- and 5 disyllabic pseudowords) which were presented via headphones. Children were asked to repeat each pseudoword first and then to pronounce it without a specified phoneme (e.g., “/folt/ without /t/”). Any pseudoword that children could not pronounce correctly was played again up to two times. Items that were still not repeated correctly were excluded from analysis (about 9% of the items). The ratio of correct responses to the total number of responses was taken into account. Cronbach’s alpha was .70.

**Rapid automatized naming (RAN).** Standard paradigms of RAN-objects and RAN-digits (Denckla & Rudel, 1976) were presented. Both conditions required to name a matrix of 40 items as quickly and accurately as possible. Simple pictured objects and digits were presented on separate sheets in five columns and eight lines. Item order was randomized and each item was presented once in each line. Children were familiarized with each condition with a 3 x 5 RAN array format. The time needed to name the full item set and any occurring errors were recorded and transformed into items named correctly per second. The correlation between conditions was .42, which corresponds to earlier studies (van den Bos, Zijlstra, & lutje Spelberg, 2002).

**ADHD-rating**. Parents were asked to answer a standardized questionnaire (Döpfner, Görtz-Dorten, Lehmkuhl, Breuer, & Goletz, 2008) which consists of 20 items with a 4-point rating scale investigating symptoms of inattention (9 items), hyperactivity (7 items) and impulsivity (4 items). A high score on the questionnaire is indicative of high ADHD symptoms.

References

van den Bos, K. P., Zijlstra, B. J., & lutje Spelberg, H. C. (2002). Life-span data on continuous-naming speeds of numbers, letters, colors, and pictured objects, and word-reading speed. *Scientific Studies of Reading*, *6*, 25–49.
