# Supplementary figures and images for: White matter alterations and tract lateralization in children with dyslexia and isolated spelling deficits
Source: Hum Brain Mapp. 2018 Sep 29;40(3):765–76. doi: 10.1002/hbm.24410 (PMC6492145; doi:10.1002/hbm.24410)

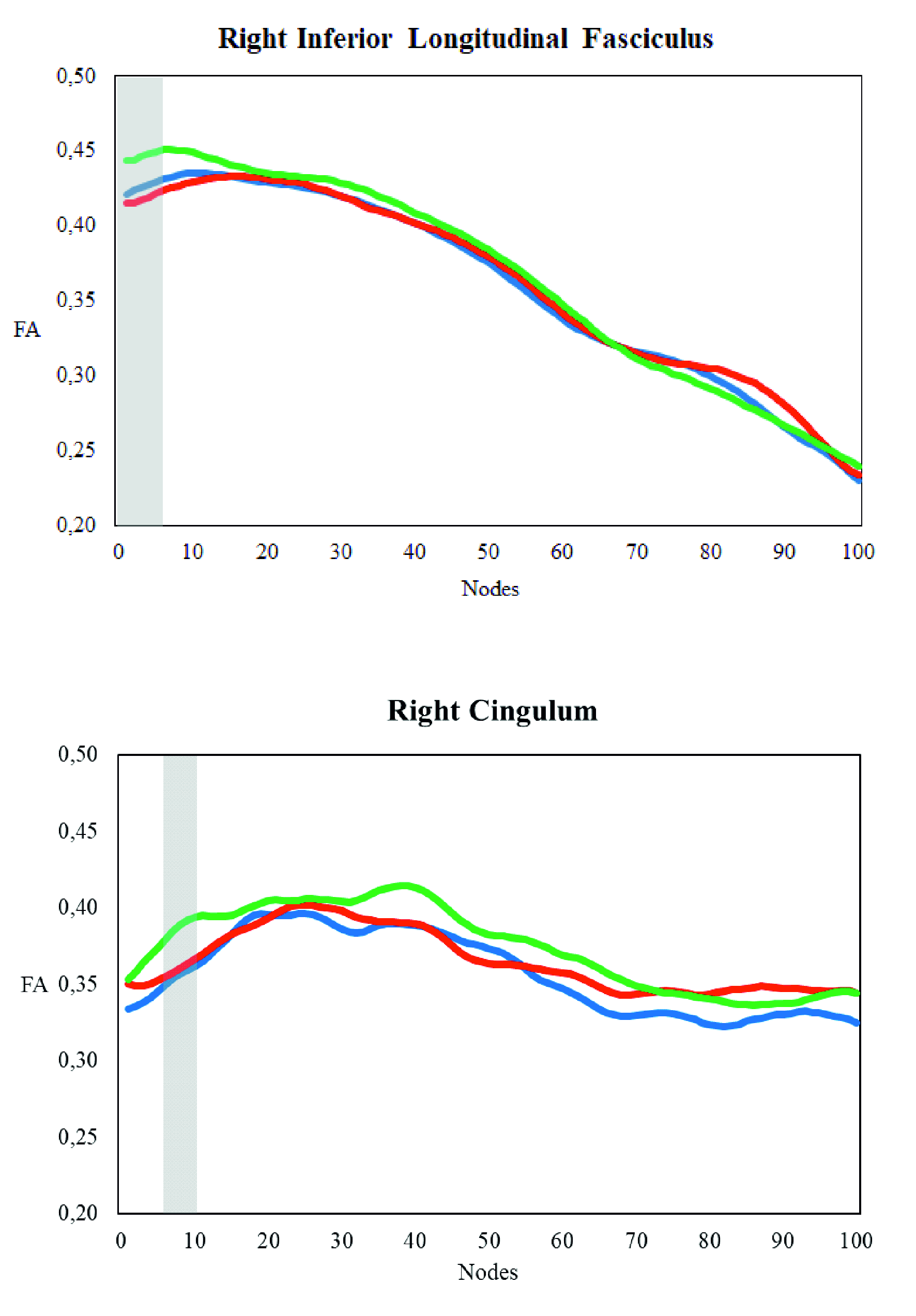

Supplement: Supplementary file 2 — Supplementary Figure S1: Tract profiles for the right inferior longitudinal fasciculus and cingulum in the three groups (blue: Typical readers and spellers; Green: Dyslexia group; Red: SD group). The gray‐shadowed areas highlight regions on the tracts where groups differed. Nodes are ordered in the posterior–anterior direction [file HBM-40-765-s002.tif]
