# Supplementary material for: White matter alterations and tract lateralization in children with dyslexia and isolated spelling deficits
Source: Hum Brain Mapp. 2018 Sep 29;40(3):765–76. doi: 10.1002/hbm.24410 (PMC6492145; doi:10.1002/hbm.24410)
Supplement: Supplementary file 3 — Supplementary Table S1: Nodes on white matter tracts were groups differed on FA. Results at the more stringent significance level of p ≤ .017 are reported in the upper part of the table. Results at p ≤ .05 are reported in the bottom part of the table. Means (M) and standard deviations (SD) on FA are reported, with the statistical significance of relevant Bonferroni‐corrected post‐hoc comparisons. [file HBM-40-765-s003.docx]

**Supplementary Table S1**

*Nodes on white matter tracts were groups differed on FA. Results at the more stringent significance level of p ≤ .017 are reported in the upper part of the table. Results at p ≤ .05 are reported in the bottom part of the table. Means (M) and standard deviations (SD) on FA are reported, with the statistical significance of relevant Bonferroni-corrected post-hoc comparisons.*

| **Significant results at *p* ≤ .017** | | | | | | | | | | | | |
| --- | --- | --- | --- | --- | --- | --- | --- | --- | --- | --- | --- | --- |
|  |  | TD | | Dyslexia | | SD | |  |  |  |  |  |
| Tract | Node | *M* | *SD* | *M* | *SD* | *M* | *SD* | *F* | *p* | *ES* | relevant contrast | *p* |
| L ILF | 54 | .365 | .024 | .391 | .036 | .381 | .032 | 4.394 | .016 | .119 | dyslexia > TD | .015 |
|  | 55 | .360 | .024 | .387 | .035 | .377 | .032 | 4.755 | .012 | .128 | dyslexia > TD | .011 |
|  | 56 | .356 | .024 | .383 | .034 | .372 | .031 | 4.831 | .011 | .129 | dyslexia > TD | .010 |
|  | 57 | .352 | .024 | .378 | .032 | .368 | .031 | 4.768 | .012 | .128 | dyslexia > TD | .011 |
|  | 58 | .348 | .024 | .373 | .031 | .364 | .032 | 4.586 | .014 | .124 | dyslexia > TD | .013 |
| R SLF | 89 | .387 | .028 | .407 | .035 | .375 | .036 | 4.379 | .017 | .133 | dyslexia > SD | .014 |
|  | 90 | .385 | .027 | .405 | .035 | .373 | .035 | 4.432 | .016 | .135 | dyslexia > SD | .014 |
|  | 91 | .384 | .027 | .403 | .034 | .371 | .035 | 4.413 | .017 | .134 | dyslexia > SD | .014 |
| **Significant results at *p* ≤ .05** | | | | | | | | | | | | |
| L ILF | 52 | .375 | .024 | .398 | .038 | .390 | .035 | 3.184 | .048 | .089 | dyslexia > TD | .050 |
|  | 53 | .370 | .024 | .395 | .037 | .386 | .033 | 3.790 | .028 | .104 | dyslexia > TD | .027 |
|  | 54 | .365 | .024 | .391 | .036 | .381 | .032 | 4.394 | .016 | .119 | dyslexia > TD | .015 |
|  | 55 | .360 | .024 | .387 | .035 | .377 | .032 | 4.755 | .012 | .128 | dyslexia > TD | .011 |
|  | 56 | .356 | .024 | .383 | .034 | .372 | .031 | 4.831 | .011 | .129 | dyslexia > TD | .010 |
|  | 57 | .352 | .024 | .378 | .032 | .368 | .031 | 4.768 | .012 | .128 | dyslexia > TD | .011 |
|  | 58 | .348 | .024 | .373 | .031 | .364 | .032 | 4.586 | .014 | .124 | dyslexia > TD | .013 |
|  | 59 | .345 | .024 | .368 | .031 | .360 | .033 | 4.294 | .018 | .117 | dyslexia > TD | .018 |
|  | 60 | .342 | .024 | .364 | .030 | .357 | .033 | 3.794 | .028 | .105 | dyslexia > TD | .029 |
|  | 61 | .339 | .025 | .359 | .030 | .353 | .034 | 3.252 | .045 | .091 | dyslexia > TD | .050 |
| R CING | 7 | .354 | .049 | .385 | .043 | .357 | .035 | 3.394 | .040 | .096 | dyslexia > TD | .051 |
|  | 8 | .357 | .049 | .389 | .043 | .360 | .035 | 3.604 | .033 | .101 | dyslexia > TD | .043 |
|  | 9 | .359 | .048 | .392 | .044 | .364 | .037 | 3.540 | .035 | .100 | dyslexia > TD | .044 |
|  | 10 | .362 | .046 | .394 | .043 | .366 | .040 | 3.346 | .041 | .095 | dyslexia > TD | .052 |
| R ILF | 1 | .421 | .035 | .443 | .037 | .415 | .036 | 3.500 | .036 | .097 | dyslexia > SD | .044 |
|  | 2 | .423 | .035 | .444 | .037 | .416 | .036 | 3.409 | .039 | .095 | dyslexia > SD | .042 |
|  | 3 | .426 | .035 | .446 | .037 | .417 | .035 | 3.540 | .035 | .098 | dyslexia > SD | .036 |
|  | 4 | .428 | .036 | .448 | .037 | .419 | .034 | 3.627 | .032 | .100 | dyslexia > SD | .034 |
|  | 5 | .430 | .036 | .450 | .037 | .421 | .033 | 3.543 | .035 | .098 | dyslexia > SD | .037 |
|  | 6 | .431 | .037 | .451 | .037 | .424 | .032 | 3.358 | .041 | .094 | dyslexia > SD | .045 |
| R SLF | 83 | .396 | .033 | .416 | .036 | .385 | .039 | 3.369 | .041 | .106 | dyslexia > SD | .039 |
|  | 84 | .395 | .032 | .415 | .036 | .383 | .038 | 3.618 | .033 | .113 | dyslexia > SD | .031 |
|  | 85 | .393 | .031 | .414 | .036 | .382 | .038 | 3.829 | .028 | .118 | dyslexia > SD | .025 |
|  | 86 | .392 | .030 | .412 | .036 | .380 | .037 | 4.003 | .024 | .123 | dyslexia > SD | .021 |
|  | 87 | .390 | .029 | .411 | .036 | .378 | .037 | 4.157 | .021 | .127 | dyslexia > SD | .018 |
|  | 88 | .389 | .028 | .409 | .036 | .376 | .036 | 4.283 | .018 | .131 | dyslexia > SD | .016 |
|  | 89 | .387 | .028 | .407 | .035 | .375 | .036 | 4.379 | .017 | .133 | dyslexia > SD | .014 |
|  | 90 | .385 | .027 | .405 | .035 | .373 | .035 | 4.432 | .016 | .135 | dyslexia > SD | .014 |
|  | 91 | .384 | .027 | .403 | .034 | .371 | .035 | 4.413 | .017 | .134 | dyslexia > SD | .014 |
|  | 92 | .382 | .027 | .401 | .034 | .370 | .035 | 4.321 | .018 | .132 | dyslexia > SD | .015 |
|  | 93 | .381 | .027 | .399 | .033 | .368 | .035 | 4.160 | .021 | .127 | dyslexia > SD | .017 |
|  | 94 | .379 | .027 | .396 | .033 | .366 | .035 | 3.952 | .025 | .122 | dyslexia > SD | .020 |
|  | 95 | .378 | .027 | .394 | .032 | .365 | .035 | 3.721 | .030 | .115 | dyslexia > SD | .025 |
|  | 96 | .376 | .028 | .391 | .032 | .363 | .035 | 3.497 | .037 | .109 | dyslexia > SD | .032 |
|  | 97 | .374 | .028 | .389 | .031 | .362 | .035 | 3.290 | .044 | .103 | dyslexia > SD | .039 |
| L AF | 32 | .385 | .053 | .384 | .038 | .353 | .046 | 3.248 | .045 | .092 | TD > SD | .085 |
|  | 33 | .387 | .052 | .386 | .038 | .356 | .046 | 3.403 | .039 | .096 | TD > SD | .073 |
|  | 34 | .390 | .050 | .389 | .039 | .358 | .045 | 3.476 | .037 | .098 | TD > SD | .066 |
|  | 35 | .392 | .049 | .392 | .040 | .361 | .046 | 3.476 | .037 | .098 | TD > SD | .064 |
|  | 36 | .395 | .048 | .394 | .042 | .363 | .047 | 3.383 | .040 | .096 | TD > SD | .069 |
|  | 37 | .397 | .048 | .396 | .044 | .366 | .048 | 3.217 | .047 | .091 | TD > SD | .078 |
| L CING | 45 | .401 | .038 | .423 | .038 | .429 | .042 | 3.134 | .050 | .089 | SD > TD | .060 |
|  | 46 | .399 | .038 | .423 | .038 | .428 | .041 | 3.422 | .039 | .097 | SD > TD | .054 |
|  | 47 | .398 | .040 | .423 | .038 | .426 | .040 | 3.419 | .039 | .097 | SD > TD | .064 |
|  | 48 | .397 | .044 | .423 | .038 | .424 | .040 | 3.154 | .049 | .090 | SD > TD | .095 |
|  | 76 | .348 | .039 | .369 | .031 | .374 | .042 | 3.298 | .043 | .093 | SD > TD | .068 |
|  | 77 | .347 | .038 | .370 | .030 | .374 | .041 | 3.681 | .031 | .103 | SD > TD | .049 |
|  | 78 | .347 | .038 | .369 | .030 | .373 | .040 | 3.693 | .030 | .103 | SD > TD | .049 |
|  | 79 | .347 | .039 | .369 | .030 | .373 | .039 | 3.366 | .041 | .095 | SD > TD | .064 |

*Note.* L: left; R: right; ILF: inferior longitudinal fasciculus; SLF: superior longitudinal fasciculus; CING: cingulum; AF: arcuate fasciculus. Effect sizes (*ES*) are calculated as partial eta-squared.
